# Sustainable Applications of Animal Waste Proteins

**DOI:** 10.3390/polym14081601

**Published:** 2022-04-14

**Authors:** Svetlana Timorshina, Elizaveta Popova, Alexander Osmolovskiy

**Affiliations:** Department of Microbiology, Faculty of Biology, Lomonosov Moscow State University, 119991 Moscow, Russia; e.a.popova1994@gmail.com (E.P.); aosmol@mail.ru (A.O.)

**Keywords:** collagen, keratin, protein hydrolysates, biomedicine, biodegradation, sustainability

## Abstract

Currently, the growth of the global population leads to an increase in demand for agricultural products. Expanding the obtaining and consumption of food products results in a scale up in the amount of by-products formed, the development of processing methods for which is becoming an urgent task of modern science. Collagen and keratin make up a significant part of the animal origin protein waste, and the potential for their biotechnological application is almost inexhaustible. The specific fibrillar structure allows collagen and keratin to be in demand in bioengineering in various forms and formats, as a basis for obtaining hydrogels, nanoparticles and scaffolds for regenerative medicine and targeted drug delivery, films for the development of biodegradable packaging materials, etc. This review describes the variety of sustainable sources of collagen and keratin and the beneficial application multiformity of these proteins.

## 1. Introduction

Agriculture is one of the most important sectors of the economy, growing and developing each year, following the needs of humanity. The rise of the global population leads to an increase in the agricultural market volume. According to the Food and Agriculture Organization of the United Nations, world meat and fisheries together with aquaculture (excluding aquatic plants) production scaled up by 44 and 41%, respectively, between 2000 and 2019 resulting in 515 million tons [1]. The necessity of expansion in the animal husbandry production induces not only the urgency to advance methods of intensive animal farming and aquaculture, but also sustainable and profitable ways of waste processing.

Animal by-products are rich in nitrogen-containing organic compounds, including fibrillar proteins such as collagen and keratin. The first one is one of the main proteins of connective tissue extracellular matrix, the second one is the major polypeptide type of epidermis and its derivatives. The fibrillar structure of these proteins provides greater stability essential to structural and protective functions. Because of their special properties, collagen and keratin can be used in biomedicine and biotechnology to produce nanoparticles, scaffolds for cell engineering, bioplastics, bioremediation agents, as well as for the obtaining of oligopeptides and amino acids [2,3].

**Figure 1:**
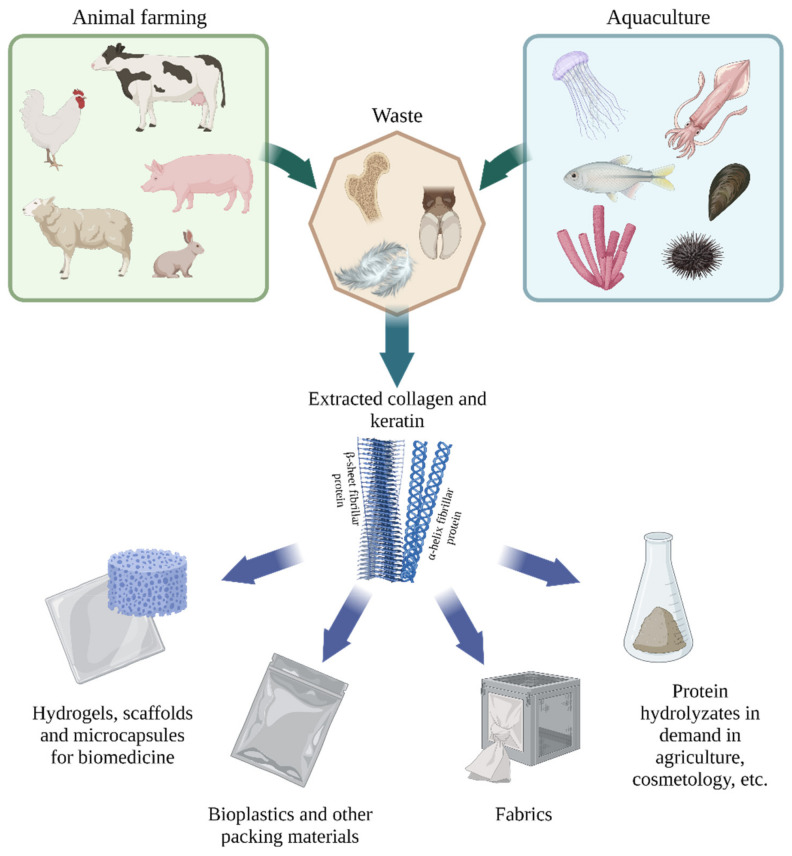
Sources and applications of collagen and keratin (created with BioRender.com, access date: 9 November 2021).

There are two main sustainable sources of these fibrillar proteins: animal farming and aquaculture waste (Figure 1). The most well-known sources of collagen and keratin are by-products of leather and meat production. Unclaimed skin trimmings, tendons, cartilage, bone tissue contain a high percentage of collagen, however, this production may be associated with the risk of the spread of bovine spongiform encephalopathy (BSE) and other prion diseases [4,5]. Marine sources of collagen are devoid of such epidemiological problems, so today a rather significant niche is occupied by the production of collagen from the wastes of fishing and fish processing industries, for example, collagen can be obtained from various commercial fish, sea urchins, jellyfish, sharks, starfish, and sponges [6].

The most saturated with keratin are livestock and poultry waste, for instance, skin, bristles, feathers. During the 2000–2019 period, world chicken meat production reached over 115 million tons, coming out on top with 35% of global meat production. Considering that feathers make up 5–7% of the total mass of domestic fowl, about 8 million tons of chicken feathers alone were formed in 2019 [1].

Currently, various disposal methods of organic waste are used. Incineration and landfilling are common, low resource and energy intensive processes. However, such approaches lead to environmental pollution, the spread of pathogens and prevent the use of agricultural by-products as a source of valuable proteins, oligopeptides and amino acids [7,8]. Chemical (acid or alkaline) hydrolysis results in the formation of free amino acids but requires subsequent neutralization of caustic reagents and purification of the target substance from unclaimed amino acid derivatives [9]. Therefore, the search and development of new sustainable ways for the animal waste recycling are in demand. The synthesis of carbon materials for biocatalysis and bioremediation by carbonization is an energy intensive and at the same time promising method for processing agricultural by-products devoid of the disadvantages described above [10,11]. Increasing the efficiency and advancing techniques for fine adjustment of carbonization are the topical tasks of modern science. It should be noted that this approach makes it impossible to use proteins and their hydrolysis products. In this connection, the focus of researchers attention is also on the development of physicochemical and enzymatic methods that allow obtaining collagen, keratin and their hydrolysates for their further applying in medicine, pharmaceuticals, cosmetology, bioremediation, the production of degradable green materials, as well as fertilizers and feed additives. The study of microorganisms synthesizing collagenolytic and keratinolytic proteases opens up new promising directions in the advancement of methods for the isolation and processing of protein polymers from animal farming and aquaculture waste that meet the trends of a sustainable economy, allowing to reduce the burden on the environment through the use of biodegradable non-hazardous agents and increment the accuracy of the target product yield because of the specificity of enzyme action [2,3].

This review is devoted to the current data on the biochemical characteristics of fibrillar proteins that may be obtained from agricultural by-products—collagen and keratin, and the possibilities of sustainable applying these substances as secondary raw materials in various fields, for example, for production of implants, degradable packaging materials, components for bioremediation.

## 2. Main Proteins of Animal Waste

The composition of living organisms includes a variety of fibrillar proteins: keratin, collagen, elastin, fibrin, etc. Blood rich in fibrinogen (fibrin precursor protein) represents up 4–7.5% of the total weight of livestock and poultry, and the protein content in the blood depends on the animal species, but rarely exceeds 30% [12]. While collagen is considered the most abundant protein in mammals, accounting for over 30% of the body’s total protein composition [13,14,15]. And keratin accounts for up to 90% of the total weight of skin derivatives, such as wool and feathers, which facilitates its extraction [12,16]. Regarding that, keratin and collagen are of particular interest for the development of methods for the large-scale biomedicine and biotechnology production of materials.

### 2.1. Keratin: Structure and Properties

Keratins are insoluble fibrillar proteins synthesized in vertebrate epithelial cells and perform structural, protective functions, and also play an important role in cell differentiation [17,18]. According to the classification formed in the last century based on X-ray analysis data, these proteins are divided into three groups: alpha-, beta- and gamma-keratins [19,20,21,22].

Alpha-keratins are found in the epidermis of all vertebrates, as well as in derivatives of the mammalian epidermis and some avian and reptilian appendages. The secondary structure of these proteins is saturated with alpha-helices. Beta-keratin molecules form beta layers coiled in helices and are included in avian and reptilian integument. Gamma-keratins are amorphous matrix proteins in which alpha-keratin filaments are submerged. However, the data of recent years forced to reconsider this state of affairs. Only alpha-keratins belong to the superfamily of intermediate filament (IF) proteins [23]. The ability of alpha-keratins to change from alpha- to beta-conformation has been shown under certain physical influences (heating, mechanical stress), which allows them to also be called “beta-keratins” [24]. Thus, only alpha- or IF-keratins are now classified as true keratins, and beta-keratins are proposed to be called corneous beta-proteins (CBPs) or keratin-associated beta-proteins [25,26,27]. According to the latest data, proteins previously known as gamma-keratin (now more often keratin-associated proteins (KAPs)) are inhering to different groups and not a homogeneous glassy material but have well-defined grainy structure [28]. CBPs and some KAPs (e.g., filaggrin, trichohyalin) are encoded by genes belonging to epidermal differentiation complex, which also confirms the absence of a phylogenetic relationship with IF-keratins [27,29].

Despite changes in the researchers’ views on the classification of keratinizing tissue proteins, they remain promising as biodegradable and biocompatible materials for tissue engineering, implant manufacturing, and the development of controlled drug release methods and also as a basis for the manufacture of green bioplastics. The keratins advantages include not only their biochemical and physicochemical properties but also the low cost of the substrates from which these proteins are extracted—agricultural waste. In this regard, the structure of the keratins that make up wool and feathers will be considered further.

#### 2.1.1. Keratins of Wool

**Figure 2:**
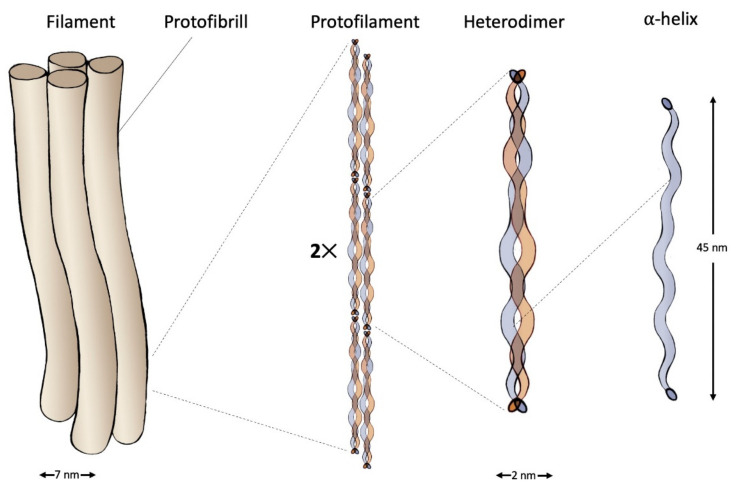
Structure of an intermediate filament composed of IF-keratin.

Wool keratins are divided into two fractions: low-sulfur that represented by intermediate filaments, and high-sulfur or KAPs. Two groups of IF-keratins exist: type I (acidic keratins) and type II (basic keratins). The molecular weight of these proteins ranges from 40 to 70 kDa. Their structure contains the C- and N-terminal domains, as well as the alpha-helical rod domain. Molecules of type I and type II keratins form heterodimers with left-handed coiled-coil structure maintained by the presence of heptad repeats rich in apolar amino acids such as leucine, isoleucine and valine in the rod domain. The mature intermediate filament consists of 4 protofibrils, each of which contains 2 protofilaments including 2 keratin heterodimers [23,30,31] (Figure 2).

C- and N-terminal domains of wool keratins are involved in the formation of disulfide bonds with matrix proteins. KAPs prevailing in the matrix are rich in amino acids, such as cysteine, proline, tyrosine, and glycine [32]. Disulfide bonds and other structural features make wool keratin a durable material suitable for biomedical application.

#### 2.1.2. Keratins of Feathers

**Figure 3:**
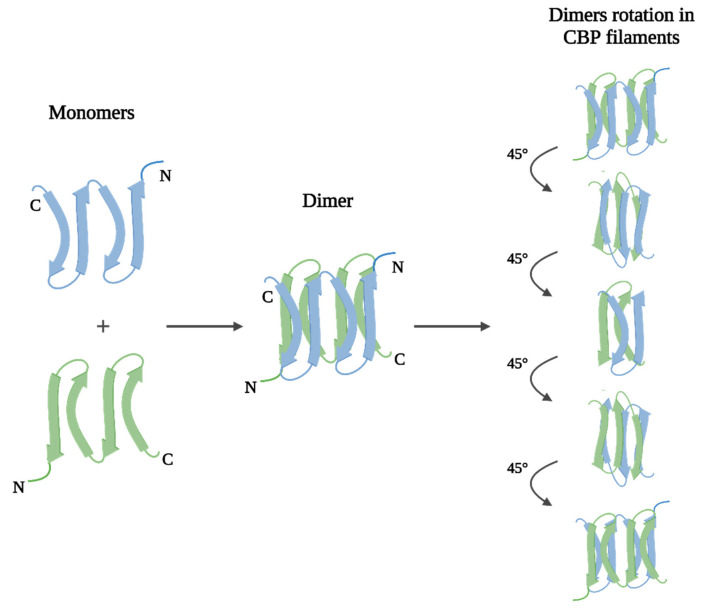
Filament structure of corneous beta-proteins (created with BioRender.com, access date: 9 November 2021).

As noted earlier, both keratins and CBPs can be found in derivatives of the epidermis of birds and reptiles, but beta proteins predominate, including in feathers. CBPs have a molecular weight from 8 to 25 kDa and, like IF-keratins, consist of three main domains: the central, N- and C-terminal. However, unlike true keratins, the molecules of CBPs are both filament- and matrix-forming at the same time. The central domain, which comprises a homologous for all Archosaurs and Squamates sequence of 34 amino acid residues, usually forms an antiparallel (more stable than parallel) beta-sheet of four beta-strands. Beta-sheet dimers constitute a helical structure filament due to a rotation of approximately 45° [33,34,35], (Figure 3).

The functions of the matrix are performed by the terminal domains of CBPs. But most likely the N-terminal domain plays the key role in the formation of intra- and intermolecular bonds, since it is, like wool KAPs, is rich in cysteine, as well as proline, tyrosine, and glycine [36].

Both wool keratins and feather beta-proteins are promising to use in biomedicine and biotechnology because of their stability, high level of structuredness, biocompatibility, as well as a combination of properties such as biodegradability and resistance to most enzymes. In recent decades, numerous research works have been devoted to the use of these proteins as bases for hydrogels, membranes, microcapsules, which are in demand for the development of treatment methods of many diseases, and bioplastics for production of green packing materials.

### 2.2. Collagen: Structure and Properties

Collagen is a fibrillar protein that is the main component of the extracellular matrix of connective tissue [37]. It is mainly localized in the ligaments, skin and tendons, and is also present in the cornea, blood vessels, bones, cartilage, intervertebral discs and intestines.

In 1955, Ramachandran and Kartha [38], based on the diffraction pattern of tendon fibers isolated from the tail of a kangaroo, showed for the first time the three-dimensional structure of collagen (Madras model). Later that year, Rich and Crick [39] determined the same triple helical structure with more stringent stereochemical criteria.

**Figure 4:**
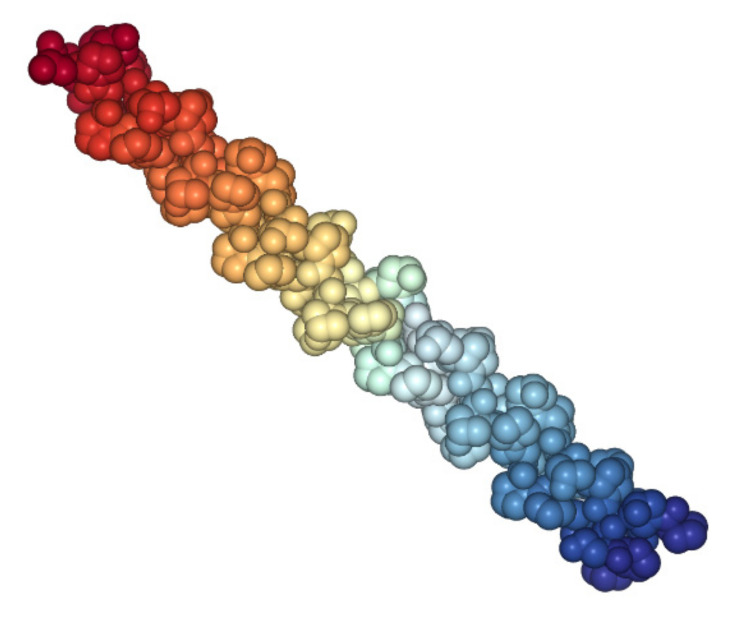
Crystal structure of tropocollagen from Protein Data Bank (PDB), 1cag [40].

Both models state that the conformation supercoils formed by three polypeptide chains. Triple helix—a unique structure (Figure 4), which is the most characteristic feature of the collagen molecule, is formed by three identical or non-identical polypeptide chains.

Each chain consists of about 1000 amino acids (sometimes more in some types of collagen), three left-handed α-chains make up an extended right-handed helix called tropocollagen. The tight packing of α-chains in tropocollagen requires that every third amino acid is a glycine, resulting in a repeating XaaYaaGly sequence, where Xaa and Yaa can be any amino acid [41]. This structure is common to all collagen types, although it can be disrupted in specific locations within the triple helix of non-fibrillar collagens [42]. The amino acids at the Xaa and Yaa positions are often proline (Pro, 28%) and 4-hydroxyproline (Hyp, 38%), respectively. ProHypGly is the most abundant triplet (10.5%) in collagen [43].

Modern sources often describe 29 types of known collagens [44], (Table 1).

However, for epidermal collagen XXIX, discovered in 2007, it was later shown that the COL29A1 gene is identical to the COL6A5 gene, and the α1(XXIX) chain corresponds to the α5(VI) chain [47]. Type I collagen is the most well studied at the moment and is also the main fibrillar protein of connective tissue, about 70% of the total amount of collagen in the body is represented by type I collagen, therefore it is the most common collagen used for the production of various biomaterials [48].

## 3. Collagen and Keratin Applications

Current trends in the transition to the use of green materials in all areas of human life are pushing scientists to search for new polymers that would replace petrochemical-based ones, be sustainable, but not inferior in strength, elasticity, and wear resistance. Collagen and keratin are one such alternative. In addition to their biochemical and physical properties, their availability and low cost of the material from which these proteins are obtained—animal farming and aquaculture waste, are also important. Fibrillar proteins can be extracted from agricultural by-products using various methods, such as chemical, thermal, and enzymatic hydrolysis, steam explosion and microwave technique [2,6,49]. Each of these approaches has some advantages and disadvantages and provides proteins with certain characteristics that meet the needs of different economy areas. For example, collagen and keratin find wide application in biomedicine due to its properties such as porosity, strength, and high biocompatibility [50,51,52,53,54].

### 3.1. Wounds and Burns Treatment

The materials development for the wounds and burns treatment is one of the urgent tasks of science. The polymers must maintain a humid environment, have absorptive and gas exchange capacity, be biocompatible, and stimulate skin protein synthesis and cell proliferation and migration.

Collagen sponges are an insoluble form of protein obtained by lyophilization of raw collagen, the porosity of the sponges depends on freezing speed and the amount of dry collagen. The ability of collagen sponges to absorb liquids, as well as high adhesive properties, allows them to protect the wound bed from drying out, mechanical trauma and bacterial infections. Collagen sponges can be enriched with elastin, glycosaminoglycans, or fibronectin to increase elasticity [55]. Collagen sponges can also be used as drug delivery system, by wetting them with antibiotic solutions [56], or by binding them with antioxidants [57].

Keratin materials can also be used to treat burns and wounds, including complicated by diabetes [58,59]. The ability of keratin to stimulate the expression of types IV and VII collagen, the proliferation and migration of fibroblasts and keratinocytes, combined with the possibility to saturate protein materials with medicinal and antimicrobial substances, is an indicator of the promise of keratin applying for biomedicine [60,61,62,63,64,65]. It was also shown that it is probably to develop injectable keratin hydrogels forming in situ and repeat the shape of the wound, which increases the quality of treatment [66,67]. In addition to native keratin hydrogels and sponges, recombinant human hair keratins nanoparticles, which turned out to be non-toxic, are effective in the treatment of wounds [68]. There are also reports of the advantages of keratin hydrogels applying in the epidermolysis bullosa [69,70], but there is no exact confirmation [71]. Improving the properties of keratin hydrogels and sponges (stability incrementing, drug release time expansion, etc.) is possible not only by changing the preparation conditions but also by synthesizing composite materials, such as keratin/alginate and keratin/chitosan [72,73,74,75,76,77].

Keratin gels and nanoparticles also exhibit hemostatic properties, reducing bleeding time and blood loss in tail amputation and liver puncture models in rats [78,79]. Cheng et al. showed the possibility of developing an oral keratin hydrogel for the treatment of gastric ulcers. The high-viscosity gel reduced bleeding, formed a barrier against gastric juice and scaffold for tissue regeneration on the surface of the wound [80].

### 3.2. Tissue Engineering

The development of materials suitable for cultivation of human cell cultures and implantation into the body is an acute problem of modern tissue engineering aimed at the progress of regenerative medicine. Currently, many scaffolds from various biological and synthetic materials have been proposed. An important place among them is occupied by collagen materials, they satisfy the basic requirements of tissue engineering—ensuring cell attachment and growth, biocompatibility and the absence of inflammatory and dystrophic tissue reactions. Also, collagen materials are successfully replaced by the body’s own tissues. Biomaterials (sponges, films, hydrogels) are used for tissue engineering or material for surgery; to solve the problem of insufficient strength of matrices, hybrid scaffolds can be used, which are composites of collagen and synthetic or natural polymers. In bone tissue engineering, for example, hybrid scaffolds based on collagen/chitosan [81], collagen/polycaprolactone [82], collagen/silk fibroin [83], collagen/hydroxyapatite [84], collagen/bioactive glass [85]. Collagen biomaterials are also used in tissue engineering of skin, cartilage, and vascular tissues [86].

Porosity, wettability, swelling property, biocompatibility, biodegradability and mechanical characteristics also allow the use of keratin materials for regenerative medicine. Like collagen, keratin can be used in various areas of tissue engineering (Table 2).

Monocomponent and composite keratin materials show greater adhesion to cells than artificial materials, and greater hardness and stability than other natural materials, including collagen, which indicates the promise of using animal waste as a source of proteins for the manufacture of scaffolds and hydrogels for tissue engineering [111,112,113,114,115]. It is worth noting that there is evidence of the benefits of hydrogels based on feather keratin over wool ones, on which most of the current research is focused [116].

### 3.3. Drug Delivery Systems

The idea of using collagen as a carrier for targeted drug delivery is not new. Back in 1995, collagen mini-pellets were developed for the delivery of proteins, like interferon [117]. The topic was continued, so in the study published in 2001 the efficacy of using collagen mini-pellets as a delivery vehicle for a single dose of tetanus toxoid (TT) and diphtheria toxoid (DT) vaccine [118] was shown.

Collagen nanoparticles are also used for targeted drug delivery; they have a high adsorption capacity and small size. It was shown that collagen nanoparticles can be used to deliver anticancer therapeutics to tumor cells [119]. Complex nanoparticles containing gold (Au), hydroxyapatite and collagen have been created to load and release doxorubicin drug [120].

Collagen films are also effective as drug delivery systems; thin, strong and transparent, most often with a complex composition. They are mainly used external in wound and burn therapy, for example, chitosan/collagen films used for the delivery of anesthetic, or PVA/collagen composite films with incorporated nanoparticles of curcumin [121,122].

Collagen hydrogels are three-dimensional cross-linked networks that can absorb and retain a significant amount of water without dissolving or losing their properties [123], they are also used for targeted drug delivery, for example in ophthalmology [124]. To improve the mechanical properties of the gel and increase its capacity, composite platforms based on collagen hydrogel have been developed [125].

Biocompatibility and biodegradability of keratin hydrogels, as well as the ability to fine-tune its microarchitecture and hence control the release time of therapeutic substances, indicate the possibility of using keratin in drug delivery systems [126,127,128]. Researchers pay special attention to the prospects for the applications of keratin materials for cancer treatment. So, many doxorubicin delivery systems have been developed based on keratin materials and composites [129,130,131,132,133,134,135]. Co-loading of docetaxel and chlorine e6 into keratin nanoparticles made it possible to achieve a synergistic effect of chemo- and photodynamic therapies [136]. Using an alginate/keratin composite contributes to the reduction of the gastrointestinal side reaction of indomethacin [137], while the mucoadhesive properties of keratin contribute to the development of systems for specific mucoadhesive drug delivery [138]. Halofuginone infused keratin gel reduced the number of postoperative adhesions formed in rats after laparotomy [139]. It has been shown that thermosensitive keratin-poly(N-isopropylacrylamide) polymers can be applied to deliver a chelating agent, deferoxamine mesylate, to an intracerebral hemorrhage lesion [140].

Keratin materials can be used not only as components of drug delivery systems, but also as a model for studying the patterns of drug penetration through the nail plate. The data of several studies indicate the promise of such a model, which makes it possible to abandon the use of native human nails and study mycoses and the effect of various pharmaceuticals due to keratin films and biomembranes [141,142,143].

### 3.4. Aesthetic Medicine and Cosmetics

Collagen is widely used in cosmetology and aesthetic medicine. Because of the ability of collagen to form films, it is used to maintain skin hydration, elasticity and prevent wrinkles in various forms—masks, gels, creams, and it can also be used in the form of injections as subcutaneous fillers [144,145].

Keratin gels can also be considered as an alternative to hyaluronic acid and can be used as fillers for skin rejuvenation [146]. Another way to apply extracted keratin is to restore the structure of damaged hair due to binding the polymer modified by the activation of thiol groups with native hair keratin [147,148].

### 3.5. Other Applications

In the food industry, collagen is used both in its native form, as a food additive [149], and in the form of collagen biomaterials. The most interesting example is edible biofilms for food packaging, such as sausages [150]. Such food films retain moisture and oxygen, preserve the organoleptic qualities of the product, and prevent fat oxidation [151]. Feather and wool keratin are also considered as an alternative to materials based on petroleum products. Keratin bioplastics are relatively strong, moisture-resistant, thermally stable, and capable of absorbing ultraviolet light, which are important qualities for packaging materials [152,153,154,155]. Encapsulation of fish oil using low-molecular-weight keratin showed good results due to the emulsifying abilities of the protein and protection of the target product from ultraviolet radiation, while the shelf life of the capsules increased with an enhanced in the percentage of keratin, the protein did not change its structure [156]. Another problem of global food security is the insufficient area of land for growing crops. Brenner and Weichold showed the possibility of cress growth on a keratin hydrogel that protects plant roots from drying out [157].

In the textile industry, collagen has been used to modify cellulose fibers, so the addition of collagen peptide to regenerated cellulose fiber has been shown to improve its properties in terms of moisture management, thermoregulation, antistatic properties, and UV protection [158]. Also, recent studies suggest using collagen to modify polyester fabric, which has been shown to improve the feel, smell, and strength of the fabric compared to conventional polyester [159].

To protect the environment, it is necessary not only to prevent the release of pollutants but also to develop methods for its adsorbing. Keratin materials have shown the ability to adsorb various dyes and heavy metals, which can be used in wastewater remediation, however, it is difficult due to the weak mechanical properties of keratin adsorbents. This problem can be solved by reinforcing keratin matrices with other polymers, such as silk fibroin or cellulose. This approach has shown effectiveness in improving the mechanical and regenerative properties of the keratin-based bioadsorbent [160,161,162,163].

Another area of animal waste proteins application is the development of strain sensors based on wool keratin alpha-helices, which have high elasticity and ability to recover, so that they can potentially be used to produce motion sensors and voice recognition systems [164].

## 4. Animal Waste Protein Hydrolysates

**Figure 5:**
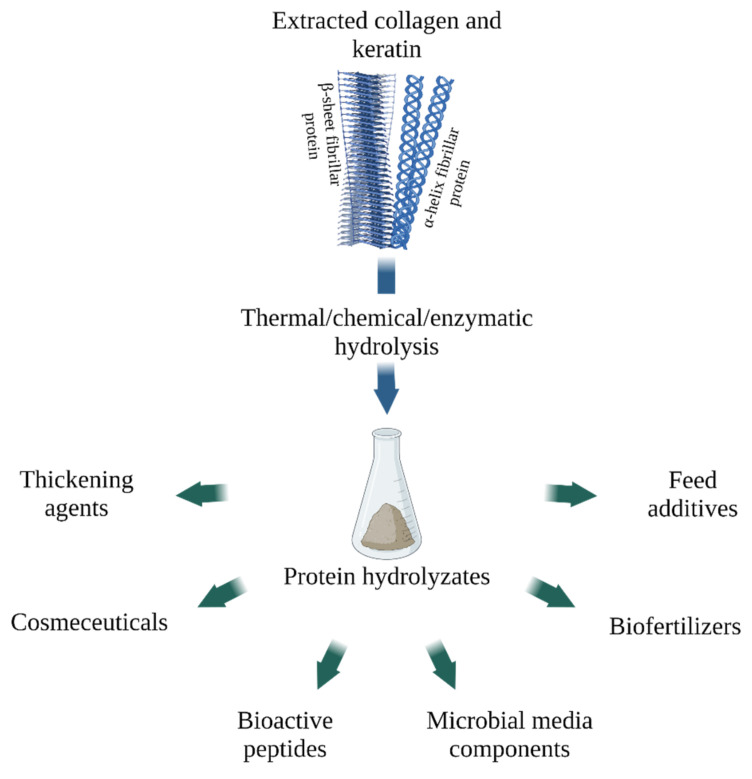
Applications of collagen and keratin hydrolysates (created with BioRender.com, access date: 9 November 2021).

Collagen and keratin hydrolysates, which include oligopeptides and amino acids, are in demand in various areas of the economy. For its obtaining, a wide range of approaches is used, for example, thermal, acidic or alkaline hydrolysis. Increasingly, however, researchers are resorting to the enzymatic production of hydrolysates, as it is a sustainable method that allows better control over the composition of the resulting mixtures [165,166], (Figure 5).

### 4.1. Gelatin and Collagen Hydrolysate

Gelatin is a heterogeneous mixture of peptides obtained from native collagen by partial hydrolysis, usually chemical, either acidic or alkaline. Further deeper enzymatic hydrolysis results in collagen hydrolysate—a mixture of short low molecular weight peptides (3–6 KDa) [167]. Collagen hydrolysate can also be derived directly from collagen, subjecting it first to thermal denaturation and then immediately to enzymatic hydrolysis [165]. For enzymatic hydrolysis, such well-known proteolytic enzymes as alcalase, pepsin, trypsin, α-chymotrypsin, neutrase, papain are usually used [167], however, microbial collagenases are also recommended by scientists for use for this purpose [168,169]. It is also possible to obtain collagen hydrolysate using acid and alkaline hydrolysis, as well as their combinations with enzymatic hydrolysis, however, using of chemical hydrolysis has several significant disadvantages, such as corrosivity and lower nutritional properties of the final product [165,170].

Gelatin is widely used in the food industry as a thickening agent, it is also used in the pharmaceutical industry for the production of soft and hard capsules, and it is used in some lithographic printing methods [171].

Collagen hydrolysate is highly bioavailable because of its small molecular size, although its final functional properties depend on the source, extraction method, and hydrolysis enzyme [165], (Table 3).

Due to its ability to increase skin hydration and elasticity, it is actively used in the cosmetic and pharmaceutical industries both externally and orally—as an external agent, it exhibits moisturizing properties and helps reduce wrinkles, and when used orally, it promotes the growth of fibroblasts and stimulates the production of new type I collagen in dermis [182,183]. It has also been shown that collagen hydrolysate improves the nutritional properties of products, promotes moisture retention in meat products, and can be used as a clarifier [165].

### 4.2. Keratin Hydrolysate

Keratin hydrolysates differ in composition depending on the way they are obtained. Mixtures of amino acids and oligopeptides of keratin origin are produced due to heat treatment, chemical and enzymatic hydrolysis [184,185,186,187]. To obtain a lower molecular weight of keratin, additional processing with ultraviolet radiation or ultrasound is possible [188,189]. However, more and more researchers are paying attention to the hydrolysis of keratin using microorganisms and their enzymes. Proteases with keratinolytic activity are a renewable and non-hazardous resource that does not require high energy costs and allows accurately predicting the yield of the target product, its quantitative and qualitative characteristics. Unfortunately, enzymatic hydrolysis is usually not efficient enough due to the high stability of keratin molecules, and therefore combined methods are being developed to produce hydrolysates using keratinases, alkalis, and reducing agents such as sulfites [190,191,192].

Keratin hydrolysates have many application fields (Table 4).

Keratin hydrolysates can be in demand in pharmaceuticals and cosmetology as components of skin care products, as they can moisturize the skin and reduce trans-epidermal water loss [193,200]. Hydrolysis of keratin can form bioactive peptides with antioxidant and chelating properties [201,202]. Literature data indicate keratin hydrolysate ability to inhibit enzymes such as angiotensin I-converting enzyme, dipeptidyl peptidase-IV, tyrosinase and matrix metalloproteinases that degrade collagen and elastin, which can be used in cosmeceuticals and pharmaceuticals including developing antihypertensive and antidiabetic substances [194,195,203].

Keratin hydrolysates are of great importance for agriculture. Mixtures of amino acids and oligopeptides can be used as biofertilizers [196,204]. It was shown that the peptides obtained due to keratin hydrolysis are able to suppress the growth of microorganisms, including phytopathogens [197]. Keratin hydrolysates have a high nutritional value and are rich in essential amino acids, especially when obtained enzymatically, which allows them to be used as feed additives [198,205].

Another area of keratin hydrolysate application is the microbial media preparation. Amino acids and oligopeptides obtained by recycling animal waste can serve as a cheap source of organic carbon and nitrogen. Many studies show that this way of reusing waste can lead to the synthesis of valuable substances, including biofuels and vitamins [199,206].

## 5. Conclusions

Thus, the importance of animal farming and aquaculture waste proteins for the development of many areas of the economy becomes obvious. Collagen and keratin are cheap substrates for the materials synthesis whose properties meet the modern requirements of various fields, including biomedicine, agriculture, food and textile industries. Recycling fibrillar proteins extracted from by-products not only reduces the cost of the product, but also scale down the burden on the environment by decreasing waste and allows enabling a shift towards rational, sustainable use of resources. The progress of techniques for the extraction and processing of collagen and keratin from organic waste will make it possible to obtain biodegradable composites with desired properties that meet the green economy trend, as well as open up new ways to use them.

## Figures and Tables

**Table 1:** Types, classes and location of collagen in tissues. Adapted from [45] with additional data from: [46].

Type	Location	Class
I	tendons, skin, artery walls, cornea, bones	Fibrillar
II	cartilage, vitreous body	Fibrillar
III	skin, vessel wall, reticular fibres, intestines, uterus	Fibrillar
IV	basal membrane, capillaries	Network-forming
V	skin, placenta, cornea, bones	Fibrillar
VI	bones, vessels, skin, cornea, cartilage	Network-forming
VII	skin, mucous membranes, bladder, umbilical cord, amniotic fluid	FACIT ^1^
VIII	skin, heart, brain, kidneys, vessels, cartilage, bones	Network-forming
IX	cornea, vitreous body, cartilage	FACIT
X	cartilage	Network-forming
XI	cartilage, intervertebral discs	Fibrillar
XII	tendons, skin, cartilage	FACIT
XIII	skeletal muscles, heart, eye, skin, endothelial cells	MACIT ^2^
XIV	skin, nerves, tendons, bones, cartilage	FACIT
XV	skin, capillary vessels, heart, ovaries, testicles, placenta, kidneys	MULTIPLEXINs ^3^
XVI	skin, heart, kidneys, smooth muscle	FACIT
XVII	hemi desmosomes in epitelia	MACIT
XVIII	liver, kidneys, lungs	MULTIPLEXINs
XIX	skin, kidneys, liver, placenta, spleen, prostate gland	FACIT
XX	corneal epithelium	FACIT
XXI	stomach, skeletal muscles, kidneys, vessels, heart, placenta	FACIT
XXII	tissue junctions	FACIT
XXIII	metastatic carcinogenic cells	MACIT
XXIV	brain, muscle, kidneys, liver, lungs, ovaries, testicles, bones	Fibrillar
XXV	brain, eye, heart, testicles	MACIT
XXVI	testicles, ovaries	FACIT
XXVII	cartilage	Fibrillar
XXVIII	skin, nerves	Network-forming
XXIX	skin	Network-forming

^1^ Fibril-associated collagens with interrupted triple helices; ^2^ Membrane-associated collagens with interrupted triple helices; ^3^ Multiple triple-helix Network-forming domains and interruptions.

**Table 2:** Application areas of keratin biomaterials in tissue engineering.

Tissue Engineering Area	Biocomposite Type	References
Nerve regeneration	Keratin hydrogels and sponges	[87,88,89,90,91,92]
	Chitosan/keratin membranes	[93]
Muscle regeneration, including cardiac ones	Keratin hydrogels	[94,95,96,97]
Bone and joint regeneration, including dental implantation	Keratin hydrogels	[98,99,100,101,102]
	Keratin-polycaprolactone composites coating calcium phosphate	[103]
	Keratin/collagen/hydroxyapatite scaffolds	[104]
	Hydroxypropyl methylcellulose crosslinked keratin scaffold, containing hydroxyapatite	[105]
	Boron- and silicon-incorporated collagen/keratin cryogels	[106]
Skin regeneration	Keratin materials	[107,108]
	Keratin/polyvinylpyrrolidone scaffold	[109]
	Guar gum ester/keratin films	[110]

**Table 3:** Characteristics and applications of collagen hydrolysates.

Sources	Extraction Method	Claimed Properties	Recommended or Current Applications	Ref. ^1^
Sea cucumber *Acaudina Molpadioides*	Enzymatic (neutrase)	Antioxidant activity	Biomedicines and functional foods	[172]
Bovine bones	Enzymatic (thermolysin-like protease A69)	Moisture-retention ability and antioxidant activity	Cosmetics, biomedicines and functional foods	[173]
Marine (commercial drug Fortigel^®^ by Gelita AG, Eberbach, Germany)	Combined chemical and enzymatic	Chondroprotective properties	Osteoarthritis treatment	[174]
Not specified (commercial drug Bodybalance^®^ by Gelita AG, Eberbach, Germany)	Combined chemical and enzymatic	Improving body composition and regional muscle strength	Biologically active additives (functional foods)	[175,176,177]
Blue shark *Prionace glauca*	Enzymatic (papain, alcalase)	Increasing effect on mRNA collagen type I expression and pro-collagen I production	Cosmetics, biomedicines and functional foods	[178]
Common carp fish *Cyprinus carpio* byproduct	Enzymatic (alcalase)	Antioxidant activity	Biomedicines and functional foods	[179]
Not specified (combined food supplement containing collagen hydrolyzate Fresubin^®^ 3.2 kcal DRINK)	Not specified	Stimulation of muscle anabolism	Functional foods	[180]
Chicken stomachs (cosmetic gel formulation)	Enzymatic (Protamex^®^ (Novozymes, Copenhagen, Denmark))	Increasing skin elasticity, decreasing skin roughness, reduction of wrinkles	Cosmetics	[181]

^1^ References.

**Table 4:** Characteristics and applications of keratin hydrolysates.

Sources	Extraction Method	Claimed Properties	Recommended or Current Applications	Ref. ^1^
Chicken feathers	Alkaline-enzymatic	Skin moisturizing ability	Cosmetics	[193]
Poultry feathers	Enzymatic (extremophilic bacteria keratinase)	Matrix metalloproteinase-1-suppressive activity	Biomedicines, cosmetics	[194]
Chicken feathers	Enzymatic (*Bacillus* sp. RCM-SSR-102 keratinase)	Antioxidant and antityrosinase activity	Biomedicines, cosmetics and functional foods	[195]
Chicken feathers	Enzymatic (bacterial keratinase)	Increasing the water holding capacity, N, carbon (C) and mineral content of the soil, enhancing seed germination and growth of plant	Biofertilizers	[196]
Sheep wool	Alkaline-enzymatic (Protamex, Esperase (Novozymes, Copenhagen, Denmark), and Valkerase (BioResource International, Durham, NC, USA)	Antimicrobial activity, plant growth stimulation	Biofertilizers	[197]
Chicken feathers	Enzymatic (*Chryseobacterium sediminis* RCM-SSR-7 keratinase)	High digestibility due to the high concentration in the composition of essential amino acids and trace elements (phosphorus, potassium, calcium and iron)	Functional foods, biofertilizers	[198]
Donkey hairs	Enzymatic (*Bacillus thuringiensis* keratinase)	Suitable amino acids composition for *Saccharomyces cerevisiae* strain ATCC 64712 to produce vitamin B-complex	Microbial media preparation	[199]
Poultry feathers	Alkaline and enzymatic (*Pseudomonas* sp. keratinase)	Complete, low-cost alternative to other microorganism culture media	Microbial media preparation	[185]

^1^ References.

## Data Availability

Not applicable.
